# Long-term patient-reported outcomes after nonoperative treatment of distal radial fractures: what CT-based gaps and step-offs can be accepted?

**DOI:** 10.1007/s00068-025-02954-z

**Published:** 2025-09-09

**Authors:** Lisanne J.M. Roelofs, Tim D. Van der Meulen, Kaj ten Duis, Sven H. van Helden, Arvid V.E. Munzebrock, Eelke Bosma, Job N. Doornberg, Joep Kraeima, Jesse B. Jupiter, Jean-Paul P.M. De Vries, Nick Assink, Frank F.A. IJpma

**Affiliations:** 1 Department of Surgery, Division of Trauma Surgery, University Medical Center Groningen, Groningen, The Netherlands; 2 Department of Surgery, Division of Trauma Surgery, Isala hospital, Zwolle, The Netherlands; 3 Medical Center Leeuwarden, Department of Surgery, Division of Trauma Surgery, Leeuwarden, The Netherlands; 4 Martini hospital, Department of Surgery, Division of Trauma Surgery, Groningen, The Netherlands; 5 Orthopaedic Trauma Service, Flinders University Medical Centre, Adelaide, Australia; 6 University Medical Center Groningen, University of Groningen, 3D lab, The Netherlands; 7 Hand and Arm Center, Department of Orthopedics, Massachusetts General Hospital, Harvard Medical School, Boston, USA; 8 Department of Surgery, Division of Vascular Surgery, University Medical Center Groningen, Groningen, The Netherlands

**Keywords:** Distal radial fracture, Nonoperative, Intra-articular, CT imaging, Patient-reported outcome, DASH

## Abstract

**Purpose:**

Nonoperative treatment of distal radial fractures is recommended for intra-articular gaps or step-offs under 2 mm; a threshold based on 40-year-old radiographic data, which is still referred to despite the transition toward Computer Tomography (CT)-imaging for assessing displacement. Controversy about treatment exists when displacement slightly exceeds 2 mm. We assessed the association between CT-based gaps and step-offs and patient-reported outcomes after nonoperative treatment, and established CT-based thresholds for fracture displacement.

**Methods:**

A multicenter retrospective cohort study was performed in 176 nonoperatively treated patients with intra-articular distal radial fractures. They completed Disability of Arm, Shoulder, and Hand (DASH) questionnaire after a mean follow-up of 10 ± 4years. The association between CT-based gap and step-off and DASH was analyzed with multivariate regression analysis, where ‘β’ represents the regression coefficient indicating the estimated change in DASH score per millimeter increase in gap or step-off. Groups with increasing displacement were created (1.Gap and step-off < 2 mm, 2.Gap = 2–4 mm and step-off < 2 mm, 3.Gap < 2 mm, step-off = 2–4 mm, 4.Gap and step-off = 2–4 mm and 5.Gap or step-off > 4 mm), and DASH-scores were compared using Mann-Whitney-U tests.

**Results:**

There was no association between DASH and gap (β = 0.66 *p* = 0.414) or step-off (β = 1.73; *p* = 0.061). The difference in DASH-score between groups 1 (gap and step-off < 2 mm) and group 2 (gap = 2–4 mm and step-off < 2 mm) was not clinically relevant (4 ± 7 vs. 7 ± 11; *p* = 0.010). Groups 3,4,5, with step-offs > 2 mm were too small for comparisons, as larger step-offs were rarely accepted clinically.

**Conclusion:**

We found no association between the CT-based gaps and step-offs and DASH in patients with nonoperatively treated distal radial fractures with intra-articular displacement around and even slightly above 2 mm. Gaps up to 4 mm and step-offs up to 2 mm are associated with good patient-reported outcomes.

**Level of evidence:**

Level III, prognostic study.

**Supplementary Information:**

The online version contains supplementary material available at 10.1007/s00068-025-02954-z.

## Introduction

Assessing the extent of fracture displacement is essential to determine an optimal treatment strategy for distal radial fractures [1–4]. Guidelines propose a combination of measurements to define fracture severity and displacement, including radial inclination, radial height, volar tilt, ulnar variance, and intraarticular gap and step-off [5–8]. Intraarticular fractures with a gap or step-off below 2 mm are considered ‘minimally displaced’, and guidelines suggest nonoperative treatment [5–8]. In clinical practice, two additional groups can be identified, namely ‘moderately displaced’ (i.e. slightly above 2 mm fracture displacement) and obviously ‘severely displaced’ fractures. The latter inevitably requires surgical intervention, while the best course of treatment in the moderately displaced group remains unclear. Unfortunately, the treatment choice is highly dependent on the country, hospital, and surgeon involved [1, 9, 10]. Since the degree of fracture displacement is important for patient counseling regarding indications for surgery and prognosis, it is crucial to assess what sizes of initial gaps and step-offs as measured on CT-scans could be accepted for nonoperative treatment in order to attain good patient-reported outcomes.

The widely cited 2 mm cut-off, proposed nearly 40 years ago by Knirk and Jupiter, was based on their finding that gaps or step-offs over 2 mm were linked to increased radiographic signs of osteoarthritis [11]. More recently, they reflected on their work and identified areas for potential improvement [12]. First, it is uncertain how gap and step-off measurements from radiographs correlate with CT-based measurements [13–20]. CT-based measurements are superior at detecting intra-articular fracture displacement and are the gold standard for evaluation of articular involvement [14–16, 21]. Secondly, radiographic gaps and step-offs were correlated to radiographic signs of osteoarthritis, but its association with patient-reported outcomes is less well established [6]. Most literature focused on clinical outcomes after surgery, highlighting residual displacement after open reduction and internal fixation instead of initial fracture displacement [17, 18, 20, 22, 23]. The few studies available on the correlation between initial fracture displacement and patient-reported outcomes after nonoperative treatment have been published many years ago, and only consider radiograph-based measurements, while studies reporting on the correlation between outcome and CT measurements are lacking [22].

This study aims to evaluate the relationship between CT-based fracture displacement and long-term patient-reported outcomes after nonoperative treatment of distal radial fractures. Therefore, we posed the following research questions: (1) What is the relationship between initial fracture displacement, measured as gap and step-off on a CT-scan, and patient-reported outcomes in patients treated nonoperatively for a distal radial fracture, at least 5 years after the injury? (2) Could a new surgical cut-off value for fracture displacement be established based on the outcome of moderately displaced distal radial fractures that slightly exceed the 2 mm threshold?

## Patients and Methods

### Study design and settings

A multicenter cross-sectional study was performed in patients who were treated nonoperatively for an intra-articular distal radial fracture between January 2005 and December 2019 at the University Medical Center Groningen, Isala Hospital, Medical Center Leeuwarden, and Martini Hospital in the Netherlands. These include two level 1 and two level 2 trauma centers, respectively.

### Participants

Adult patients (> 18 years old) with a nonoperatively treated distal radial fracture, with the availability of a diagnostic CT-scan of a displaced distal radial fracture, a minimum follow-up period of 5 years were eligible for inclusion. Included were patients who had a minimally (< 2 mm) or a moderately (slightly over 2 mm) displaced fracture that was managed nonoperatively. In general, nonoperative treatment was recommended if a gap and/or step-off less than 2 mm was observed on the CT-scan. However, in our cohort, a substantial number of patients with gaps and/or step-offs slightly exceeding 2 mm have been treated nonoperatively after shared decision-making. Patients with pathological fractures were excluded from participation.

In total, 238 adult patients met the inclusion criteria. Of these, none had pathological fractures, resulting in 238 patients being available for follow-up analysis. All eligible patients were contacted by posted mail and asked to provide informed consent and complete a validated patient questionnaire. The response rate was 74%, leaving 176 patients in our study cohort. The mean age at the time of injury was 53 ± 15 years, 36% were male, and the mean time to follow-up was 10 ± 4 years (Table 1). There were no differences in patient and fracture characteristics between the level 1 and 2 trauma centers (Supplementary information Table 6).

**Table 1:** Patient characteristics

**(n = 176) **	
Age Years ± SD*	53 ± 15
Gender: male	64 (36%)
Side of fracture: left	89 (51%)
Dominant hand (%)	88 (51%)
AO classification (%)	
23B1	8 (5%)
23B2	4 (2%)
23B3	2 (1%)
23C1	55 (31%)
23C2	28 (16%)
23C3	79 (45%)
Gap (mm) ± SD*	2.3 ± 1.1
Step-off (mm) ± SD*	0.7 ± 0.9
Radial inclination (degrees) ± SD*	22 ± 4
Radial height (mm) ± SD*	11 ± 2
Ulnar variance (mm) ± SD*	0 ± 1
Volar tilt (degrees) ± SD*	0 ± 9
Follow-up (years) ± SD*	10 ± 4

*SD=standard deviation

### CT-based gap and step-off measurements

**Fig. 1:**
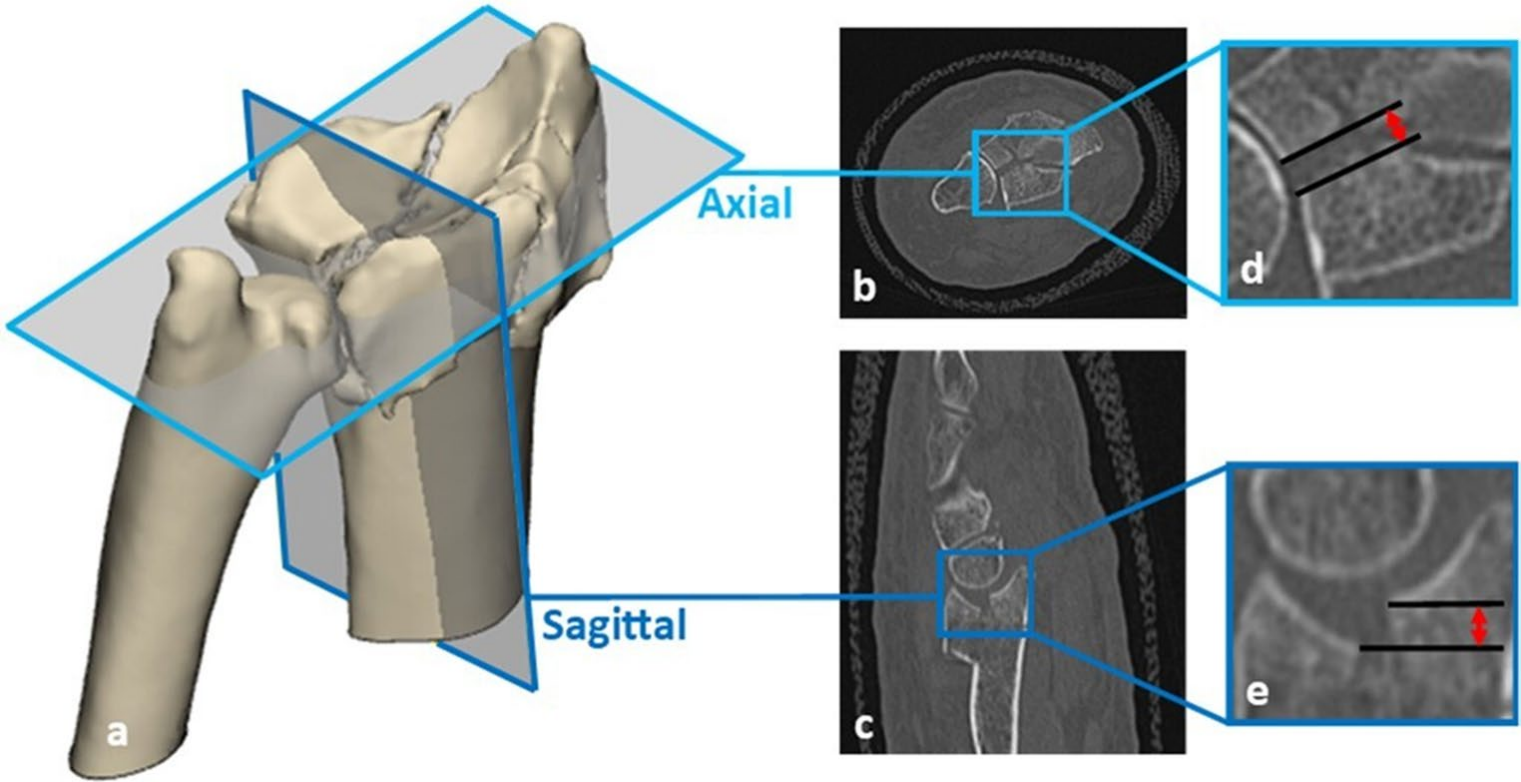
**a **3D model with the location of the captured CT-slices to illustrate the gap and step-off measurements.**b** axial CT slice, with d: detail of gap (=2.1mm) measurement parallel to the joint surface. **c** sagittal CT slice with **e** detail of step-off (=2.3mm) measurement perpendicular to the joint surface

All wrist CT-scans were reassessed for measurement and classification according to the Arbeitsgemeinschaft für Osteosynthesefragen/Orthopaedic Trauma Association (AO/OTA) classification system by two trauma surgeons (KtD, FIJ) until consensus was reached. Gap and step-off measurements were performed on the CT-scan. The fracture gap is defined as the distance between fracture fragments along the articular surface of the distal radius. The fracture step-off is the distance between fracture fragments perpendicular to the articular surface. The size of the maximum intra-articular gap and step-off displacement, measured in either the axial, coronal, or sagittal CT view, was recorded (example of the measurement method is illustrated in Fig. 1) [1]. Measurements were performed with a digital tool (accuracy 0.1 mm) in the Carestream Vue Motion imaging system of the patient files.

### Wrist radiographic measurements

**Fig. 2:**
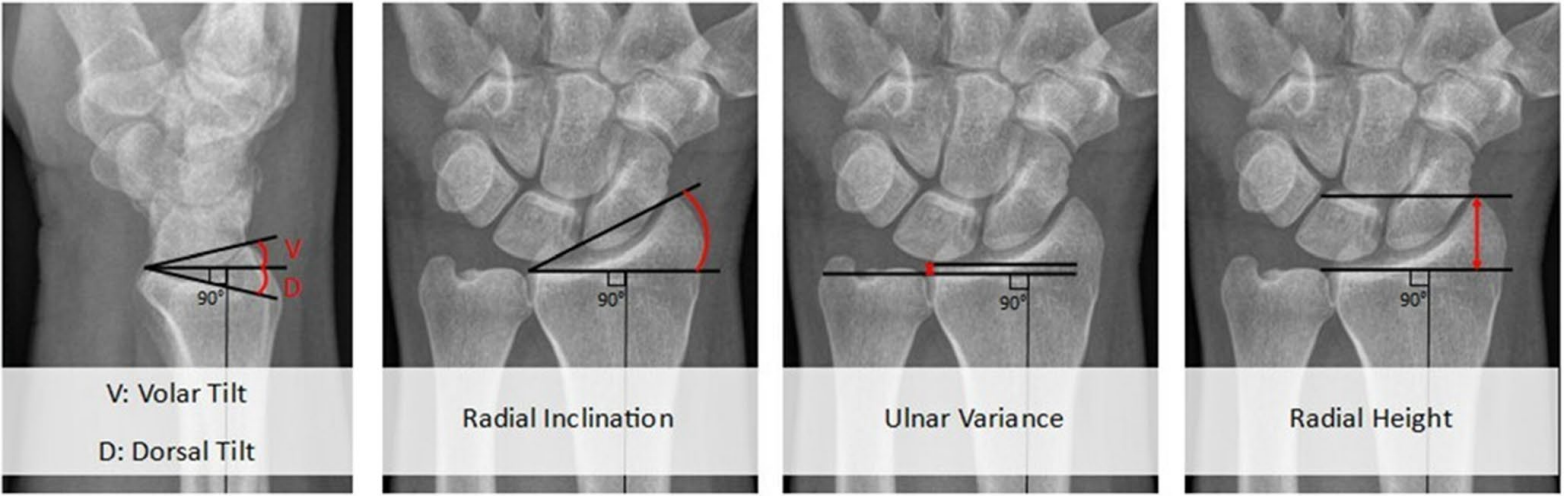
Schematic representation of radiographic measurements of an uninjured wrist. From left to right: volar tilt (normal range: 11 to 12° toward volar), radial inclination (normal range: 21 to 25 degrees), ulnar variance (normal range: −4.2 to 2.3 mm) and radial height (normal range: 8 to 14 mm) [9]

All wrist radiographs were reassessed through consensus by two consultant trauma surgeons (KtD, FIJ). Volar tilt was measured on the lateral radiograph, and radial inclination, ulnar variance and radial height were measured on the anteroposterior radiograph according to the current guidelines (Fig. 2). All measurements were based on two guiding lines; one through the axis of the radial shaft and one perpendicular to that: the ’90-degree baseline’. Volar tilt was either the volar or dorsal angulation, which is measured at the volar rim and is the angle between the 90-degree baseline and the dorsal edge of the distal radial joint. Radial inclination was the angle between the 90-degree baseline and the line between the distal radial ulnar joint (DRUJ) and radial styloid. Ulnar variance is the distance between the radial and ulnar distal articular surface at the DRUJ. Radial height is the distance between the radial articular surface and the radial styloid, measured parallel to the radial shaft midline.

### Patient-reported outcomes

All eligible patients were approached by posted mail and asked to complete the Disability of the Arm, Shoulder and Hand Questionnaire (DASH) [24]. The DASH is a validated questionnaire consisting of thirty questions used to measure a patient’s physical function and symptoms in the upper limb. The score ranges from 0 to 100, with a lower score indicating a better patient-reported outcome. The *minimum clinically important difference* (MCID) of the DASH is 10.8 and the *minimum detectable change* (MDC) is 10.8 points for the DASH [25].

### Data sources

Baseline characteristics of the participants were retrieved from the patients’ electronic records. The scores of the DASH were calculated from the patients’ responses to the surveys according to the accompanying questionnaire’s specific instructions [14].

### Primary and Secondary Study Outcomes

The primary goal of our study was to assess to which an increase in gap and/or step-off is related to worse patient-reported outcome in nonoperatively treated distal radial fractures. We therefore reassessed all CT-scans at the time of injury and determined fracture displacement using gap and step-off measurements. These measurements were correlated to validated patient-reported outcomes (DASH questionnaire) collected at a minimum time to follow-up of 5 years. Our second research goal was to assess whether a new cut-off value could be established for gaps and step-offs on distal radius CT-images. To assess the impact of gaps and steps, patients were subdivided into five groups based on the sizes of gaps and step-offs (1. Gap and step-off < 2 mm, 2. Gap 2–4 mm and step-off < 2 mm, 3. Gap < 2 mm and step-off 2–4 mm, 4. Gap and step-off 2–4 mm and 5. Gap or step-off > 4 mm). The mean DASH scores were then compared between groups to determine whether there are differences in postoperative patient complaints associated with increased gap and step-off groups.

### Ethical approval

This research plan was approved by the local ethical committee of all participating centers and the study was performed in accordance with the current guidelines and regulations (number: 201800411). This study is reported in accordance with the STROBE (Strengthening the Reporting of Observational Studies in Epidemiology) guideline.

### Data analysis and statistics

SPSS (version 28, IBM, Chicago, IL, USA) was used for statistical analysis. A multivariate regression analysis was performed to test the effect of gaps and step-offs on the DASH score, where‘β’ represents the regression coefficient indicating the estimated change in DASH score per millimeter increase in gap or step-off. The analysis accounted for potential confounders including age, gender, time to follow-up, fracture at dominant hand, accompanying ulnar fracture, radiographic fracture angulation (radial inclination, radial height, ulnar variance, volar tilt), AO/OTA type B/C3 fracture classification, and gap in case of step-off analysis and step-off in case of gap analysis.

Lastly, patients were divided into five groups based on their gap and step-off. Power analysis indicated that a sample size of at least 63 patients would be required in each group to detect a difference of 10.8 points in the DASH score, using 80% statistical power, a significance level of 0.05, and a standard deviation (SD) of 18. In groups with a sufficient number of patients (n≥63), the mean DASH scores were compared using the Mann-Whitney-U test. A p<0.05 was considered statistically significant.

### Analysis of nonparticipants

For the nonresponse analysis, we used a chi-square test for noncontinuous variables and an independent samples t-test for continuous variables. The nonresponse analysis showed no differences between responders and nonresponders in age (48 ± 17 years vs. 53 ± 15 years; p=0.079), gender (34% vs. 36% males; p=0.180) and time to follow-up (12 ± 0 vs. 10 ± 4 years; p=0.252). There was no difference in AO classification either (23B1 5% vs. 10%, 23B2 2% vs. 11%, 23B3 1% vs. 3%, 23C1 31% vs. 15%, 23C2 16% vs. 26%, 23C3 45% vs. 36%; p=0.270). There were no differences in gap (2.3 ± 2.2 vs. 2.3 ± 1.1; p=0.065), step-off (0.6 ± 0.8 vs. 0.7 ± 0.9; p=0.620), radial inclination (21 ± 7 vs. 22 ± 4; p=0.851), radial height (11 ± 3 vs. 11 ± 2; p=0.782), and palmar tilt (−3 ± 15 vs. 0 ± 9; p=0.442). There was only a small difference in ulnar variance between responders and nonresponders (0 ± 1 mm vs. 2 ± 3 mm; p<0.001), which is within the normal range.

## Results

### Relationship between initial fracture displacement and patient-reported outcome

In nonoperatively treated minimally and moderately displaced distal radial fractures, we found no clinically relevant association between the degree of fracture displacement and patient-reported outcome (Table 2 and Supplementary information Tables 4 and 5). After adjusting for potential confounders, multivariate linear regression analysis showed no association between gap and the DASH score (β=0.66; p=0.414), nor between the step-off and the DASH score (β=1.73; p=0.061), R^2^=0.178.

**Table 2:** Multivariate regression analysis of the association between gap and step-off versus DASH in minimally displaced distal radial fractures

Variable	β* (95% CI)	*P*-value
Gap	0.66 (−0.92 to 2.23)	0.414
Step-off	1.73 (−0.08 to 3.54)	0.061

Adjusting for confounders: age, gender, time to follow−up, fracture at dominant hand, accompanying ulnar fracture, radiographic fracture angulation (radial inclination, radial height, ulnar variance, volar tilt), AO/OTA type B/C3 fracture classification, and gap in case of step−off analysis and step−off in case of gap analysis.*The regression coefficient (β) indicates the expected increase in the DASH score in case the gap or step−off would increase by 1 mm

### Should the 2 mm surgical cut-off for gap and step-off be reconsidered?

**Fig. 3:**
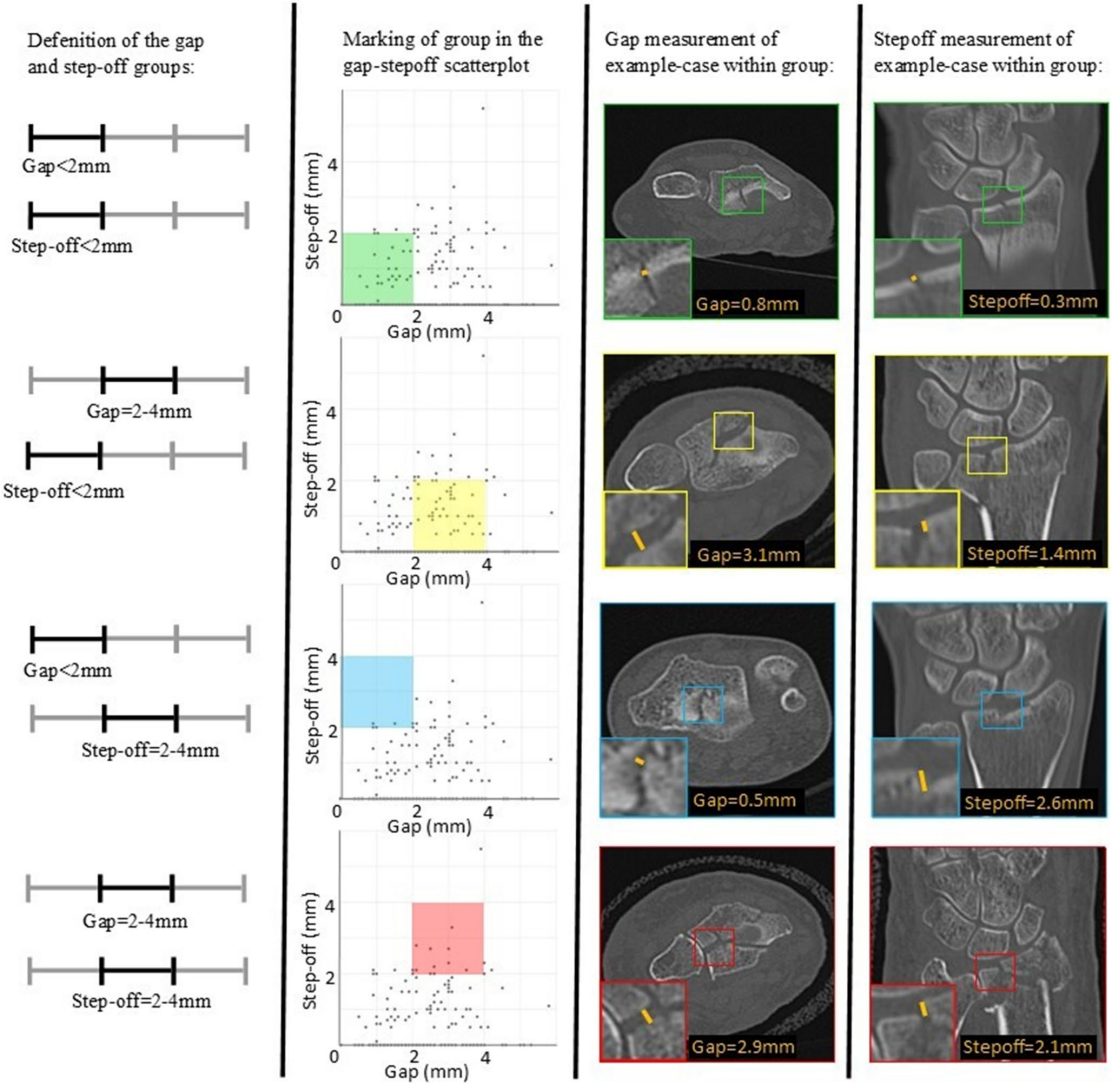
Patients were subdivided into five groups based on increasing gaps and step-offs. The scatterplots include colored areas representing subgroups based on gap and step-off cut-off values. Five groups were defined: 1. Gap and step-off < 2 mm (green), 2. Gap 2–4 mm and step-off < 2 mm (yellow), 3. Gap < 2 mm and step-off 2–4 mm (blue), 4. Gap and step-off 2–4 mm (red) and 5. Gap or step-off > 4 mm (latter not depicted)

To challenge the current surgical cut-off value of 2 mm for the intra-articular gap and step-off, patients were subdivided into five groups based on increasing gaps and step-offs (Fig 3). Overall, patients in each group reported good clinical outcomes after nonoperative treatment of a minimally or moderately displaced distal radius fracture. An increase in gap and step-off showed a slight increase in DASH score (Table 3). Groups 1 and 2 had a sufficient number of patients, while groups 3, 4, and 5 did not contain enough patients for statistical analysis, likely because physicians in clinical practice are generally reluctant to accept larger step-offs (>2mm). A comparison between groups 1 and 2 showed a difference in DASH score of only 3 points, which was below the 10.8 MCID (t-test; p=0.010).

**Table 3:** Patient-reported outcome after nonoperative treatment of distal radial fractures, stratified for increasing fracture displacement

Group	1.	2.	3.	4.	5.	*p*-value*
Gap	< 2 mm	2–4 mm	< 2 mm	2–4 mm	> 4 mm	
Step-off	< 2 mm	< 2 mm	2–4 mm	2–4 mm	> 4 mm	
N patients	71	76	4	12	13	
DASH	4 ± 7	7 ± 11	3 ± 4	12 ± 20	12 ± 11	0.010

*p−value is based on a comparison of the mean DASH score between groups 1 and 2 using a Mann−Whitney−U test

## Discussion

Conventional radiographs remain essential for the initial diagnosis of distal radius fractures and for identifying the presence of an intra-articular component. From our perspective, every patient with a suspected distal radius fracture should first be evaluated with a radiograph. If an intra-articular fracture is suspected based on the radiograph, a CT scan is performed to assess the extent of intra-articular displacement and to guide treatment planning aimed at restoring joint congruency. We therefore do not consider conventional X-rays outdated, but consider CT as a complementary tool in cases with suspected intra-articular involvement. However, the effect of CT-based gaps and step-offs on functional outcomes in patients with nonoperatively treated distal radial fractures remains disputable. As a result, it is challenging to decide on operative versus nonoperative treatment and estimate prognosis based on CT measurements, especially in moderately displaced fractures. To improve patient counseling and shared decision-making, we evaluated the association between gaps and step-offs in a cohort of nonoperatively treated patients with minimally and moderately displaced distal radial fractures. Based on our findings, patients with moderately displaced intra-articular distal radial fractures should be told that fractures with a gap up to 4 mm and a step-off up to 2 mm, as measured on a CT-scan, may also result in good long-term patient-reported outcomes after nonoperative treatment.

### Relationship between initial fracture displacement and patient-reported outcome

In patients with nonoperatively treated distal radial fractures, we found no association between the CT-based gap and step-off and patient-reported outcome (DASH score). In literature, we found that there is a lack of research into the relationship between CT-based measurements of these fractures and PROMs. As a result, many guidelines still rely on radiographic rather than CT-based measures [5–8]. However, several studies compared gaps and step-offs on radiographs and PROMs. Jaremko et al., Földhazy et al., Young et al., and Giannoudis et al. found no relationship between radiograph-based gap and step-off and patient-reported outcomes in mostly nonoperatively treated patients with minimally displaced fractures [19, 26–28]. In two other studies, radiograph-based measurements were performed to compare outcomes between an operative and a nonoperative group. Both found no association between radiograph-based gaps and step-offs on mid-term outcomes [17, 29]. In recent years, literature has shown better reliability of the CT-based measures compared to radiographs [13, 16]. Cole et al. included minimally and moderately displaced intra-articular distal radial fractures (up to 5 mm) and studied the reliability of CT and radiographic measurement of fracture displacement [13]. They demonstrated that CT-scans are more reliable than radiographs, especially in the <2mm displaced fractures [13]. Some studies do analyze the association between patient-reported outcomes and (postoperative) CT-based gap and step-off measurements, albeit after surgical interference. Four studies found no association between postoperative gap and step-off as measured on CT-images and PROMs [18, 30–32]. To our knowledge, studies that analyze the relationship between CT-based gap and step-offs in nonoperatively managed distal radial fractures are lacking. Studies mainly focus on radiographic measurements, and if CT measurements are available, these are often derived from post-operative CT. Therefore, our results cannot easily be compared with the available literature. Our current study contributes to the literature by demonstrating no relationship between the CT-based gap and step-off measurements and the DASH score in minimally and moderately displaced intra-articular distal radial fractures treated nonoperatively.

### Should the 2 mm surgical cut-off for gap and step-off be reconsidered?

We found no clinically relevant differences in PROMs between group 1 with a gap and step-off of <2mm (DASH=4) and group 2 with a step-off of <2mm and a gap of 2-4mm (DASH=7). The difference of 3 points is not noticeable for the patient (MCID=10.8) [25]. Current guidelines still refer to the over 40-year-old 2 mm surgical cut-off, despite new insights that suggest otherwise [5, 12]. In literature, there is a lack of studies on outcomes in nonoperatively treated patients with CT-based gaps and/or step-offs exceeding the 2 mm surgical cut-off. Only a few studies analyzed outcomes in patients with moderately displaced fractures with a gap and/or step-off slightly exceeding the 2 mm cut-off. Mulders et al. performed a meta-analysis to evaluate the association between fracture reduction, as determined by plain radiographs instead of CT-scans like in our study, and patient-reported outcome in patients with a displaced distal radial fracture [33]. They concluded that inadequate fracture reduction is associated with slightly worse patient-reported outcomes, however, these differences are not clinically relevant. The results from this meta-analysis, including 1961 patients, show that a limited degree of residual fracture displacement is clinically well-tolerated, but it is still unclear to what extent [33]. Additionally, the measurements were based on radiographs rather than CT-scans, making them difficult to compare with our findings. Also, Young et al. report on a few patients (8 out of 25) in whom a step-off of >2mm was accepted, of whom 5 had excellent results [27]. Unfortunately, this study is difficult to compare with ours due to a lack of standardized PROMs. Our findings contribute to the current literature by proposing a revision of the traditional 2 mm displacement threshold for distal radial fractures, established 40 years ago using radiographs, based on our cross-sectional study with a large cohort of nonoperative patients and CT-based measurements. We recommend informing patients with moderately displaced fractures opting for nonoperative treatment that gaps up to 4 mm and step-offs up to 2 mm on CT-scans are associated with good outcomes. 

### Limitations

This study has some strengths and some limitations. First of all, the retrospective study design and patient selection based on the availability of a CT-scan inevitably causes a selection bias. Unfortunately, it is not feasible to conduct a randomized study with 10 years of follow-up in patients with moderately displaced radial fractures to assess the long-term impact of small gaps and step-offs. In the early years, a CT-scan was not as commonly used in clinical practice as nowadays. CT imaging plays a crucial role in detecting malalignment that may not be visible on plain radiographs. Recently, Dankelman et al. demonstrated that up to 53% of fractures appearing well-aligned on post-reduction radiographs actually showed malalignment on CT-scans [34]. The findings of our study apply specifically to patients with distal radius fractures assessed by CT-scan and cannot be directly extrapolated to fractures evaluated solely by radiographs. Secondly, patients were selected for nonoperative treatment based on shared decision-making, including surgeon’s and patient’s preferences. In this study, it is unknown how this decision was made exactly and how the process has developed over the years. However, our nonresponse analysis showed no differences between responders and nonresponders in terms of age, gender, time to follow-up and fracture classification, and we had a high response rate of 74% at a mean follow-up of 10 ± 4 years. Moreover, we found no differences in patient and fracture characteristics between the level 1 and level 2 trauma centers. Therefore, we believe our results are generalizable. The third limitation is the unequal distribution of patients over the different gap and step-off groups, likely because physicians in clinical practice tend not to accept larger step-offs (>2mm). As a result, our data cannot determine the effect of increased step-off (i.e., 2–4mm) on patient-reported outcomes after nonoperative treatment. Overall, our data can be seen as a reflection of clinical practice, where larger step-offs are generally not accepted. This might imply an experience-based consensus among surgeons against accepting 2-4mm step-offs in clinical practice. Fourth, current literature reports some interobserver variability in the gap and step-off measurements [13, 14, 16, 35, 36]. A limitation of CT-based 2D measurements of gap and step-off in distal radius fractures is their poor to fair interobserver agreement, which arises from variability in slice selection and interpretation among observers [36]. However, evaluating the association between CT-based gap and step-off, and patient-reported outcomes remains important, as these measurements are among the most straightforward and most widely used methods for assessing joint surface incongruity [2, 14, 16].

## Conclusion

In patients with minimally and moderately displaced distal radial fractures, we found no significant relationship between the gap and step-off measurements and patient-reported outcomes at long-term follow-up. Patients with moderately displaced distal radial fractures who prefer nonoperative treatment can be informed that gaps up to 4 mm and step-offs up to 2 mm, as measured on CT-scans, are associated with favorable patient-reported outcomes at long-term follow-up. Since larger step-offs (>2 mm) are rarely accepted in clinical practice, the effect of 2–4 mm step-offs on patient-reported outcomes after nonoperative treatment remains unclear. This information can help guide patient counseling and set realistic expectations for recovery. These emerging insights from CT imaging suggest that the traditional 2 mm fracture displacement threshold, based on radiographs and set over 40 years ago, could be reconsidered. Future studies should focus on large prospective cohorts to investigate the relationship between fracture displacement and patient-reported outcomes, eventually using not only 2DCT but also innovative 3DCT, and data-driven measurement tools [36–38].

## Supplementary Information

Below is the link to the electronic supplementary material.

ESM 1 (DOCX 271 KB)

## Data Availability

No datasets were generated or analysed during the current study.

## References

[1] Lalone EA, Grewal R, King GJ, MacDermid JC. A structured review addressing the use of radiographic measures of alignment and the definition of acceptability in patients with distal radius fractures. Hand. 2015;10(4):621–38. 26568715 10.1007/s11552-015-9772-9 PMC4641087

[2] Esworthy G, Johnson N, Divall P, Dias J. Methods of assessing intra-articular distal radial fractures. Journal of Hand Surgery (European Volume). 2023;48(11):1231–2. 37387235 10.1177/17531934231184130

[3] Hunt JJ, Lumsdaine W, Attia J, Balogh ZJ. AO type‐C distal radius fractures: the influence of computed tomography on surgeon’s decision‐making. ANZ J Surg. 2013;83(9):676–8. 23088619 10.1111/j.1445-2197.2012.06311.x

[4] Neuhaus V, Bot A, Guitton T, Ring D. Influence of surgeon, patient and radiographic factors on distal radius fracture treatment. Journal of Hand Surgery (European Volume). 2015;40(8):796–804. 25342650 10.1177/1753193414555284

[5] Hannemann PFW, Schep NWL, Vos DI, Deijkers RLM, Colaris JW. J. vL. Distale radiusfracturen: Richtlijnen 2021 [2024 aug 14]. Available from: https://richtlijnendatabase.nl/richtlijn/distale_radiusfracturen/startpagina_-_distale_radiusfracturen.html

[6] American_Academy_of_Orthopaedic_Surgeons. The treatment of distal radius fractures: guideline and evidence report. Adopted by the America n Academy of Orthopaedic Surgeons Board of Directors. 2020.

[7] Chen NC, Jupiter JB. Management of distal radial fractures. The Journal of Bone and Joint Surgery-American Volume. 2007;89(9):2051–62. 17768207 10.2106/JBJS.G.00020

[8] Ilyas AM, Jupiter JB. Distal radius fractures—classification of treatment and indications for surgery. Orthop Clin North Am. 2007;38(2):167–73. 17560399 10.1016/j.ocl.2007.01.002

[9] Mulders MA, Rikli D, Goslings J, Schep N. Classification and treatment of distal radius fractures: a survey among orthopaedic trauma surgeons and residents. Eur J Trauma Emerg Surg. 2017;43(2):239–48. 26872680 10.1007/s00068-016-0635-z PMC5378748

[10] Doermann A, Gupta DK, Wright DJ, Shafiq B, Hacquebord J, Rafijah G, et al. Distal radius fracture management: surgeon factors markedly influence decision making. JAAOS: Global Research and Reviews. 2023;7(3):e23. 10.5435/JAAOSGlobal-D-23-00002 PMC9984156 36867522

[11] Knirk JL, Jupiter JB. Intra-articular fractures of the distal end of the radius in young adults. JBJS. 1986;68(5):647–59. 3722221

[12] Haus BM, Jupiter JB. Intra-articular fractures of the distal end of the radius in young adults: reexamined as evidence-based and outcomes medicine. The Journal of Bone and Joint Surgery-American Volume. 2009;91(12):2984–91. 19952264 10.2106/JBJS.I.00269

[13] Cole RJ, Bindra RR, Evanoff BA, Gilula LA, Yamaguchi K, Gelberman RH. Radiographic evaluation of osseous displacement following intra-articular fractures of the distal radius: reliability of plain radiography versus computed tomography. J Hand Surg. 1997;22(5):792–800. 10.1016/s0363-5023(97)80071-8 9330135

[14] Arora S, Grover SB, Batra S, Sharma VK. Comparative evaluation of postreduction intra-articular distal radial fractures by radiographs and multidetector computed tomography. The Journal of Bone and Joint Surgery-American Volume. 2010;92(15):2523–32. 21048172 10.2106/JBJS.I.01617

[15] Nascimento VG, da Costa AC, Falcochio DF, Lanzarin LD, Checchia SL, Chakkour I. Computed tomography’s influence on the classifications and treatment of the distal radius fractures. Hand. 2015;10(4):663–9. 26568720 10.1007/s11552-015-9773-8 PMC4641105

[16] Gong X, An G, Gao Z, Li S, Rong G. The role of CT in the diagnosis and treatment of distal radius fracture. Zhonghua Wai Ke Za Zhi. [Chinese J Surgery]. 2006;44(20):1414–6. 17217837

[17] Lalone E, MacDermid J, Grewal R, King G. Patient reported pain and disability following a distal radius fracture: a prospective study. Open Orthop J. 2017;11:589. 28979578 10.2174/1874325001711010589 PMC5620403

[18] Teunis T, Meijer S, van Leeuwen W, Jupiter J, Rikli D. Are radiographic characteristics associated with outcome in surgically treated distal radius fractures?? J Hand Surg. 2023;48(1):84. e1-. e13. 10.1016/j.jhsa.2021.09.020 34794848

[19] Jaremko J, Lambert R, Rowe B, Johnson J, Majumdar S. Do radiographic indices of distal radius fracture reduction predict outcomes in older adults receiving Conservative treatment? Clin Radiol. 2007;62(1):65–72. 17145266 10.1016/j.crad.2006.08.013

[20] Teunis T, Jupiter J, Schaser K, Fronhöfer G, Babst R, Langer M, et al. Evaluation of radiographic fracture position 1 year after variable angle locking volar distal radius plating: a prospective multicentre case series. J Hand Surg (European Volume). 2017;42(5):493–500. 10.1177/1753193417690478 28181454

[21] Larouche J, Pike J, Slobogean GP, Guy P, Broekhuyse H, O’Brien P, et al. Determinants of functional outcome in distal radius fractures in high-functioning patients older than 55 years. J Orthop Trauma. 2016;30(8):445–9. 26978132 10.1097/BOT.0000000000000566

[22] Jupiter JB, Marent-Huber M. Operative management of distal radial fractures with 2.4-millimeter locking plates: a multicenter prospective case series. JBJS. 2009;91(1):55–65. 10.2106/JBJS.G.01498 19122079

[23] Trumble TE, Schmitt SR, Vedder NB. Factors affecting functional outcome of displaced intra-articular distal radius fractures. J Hand Surg. 1994;19(2):325–40. 10.1016/0363-5023(94)90028-0 8201203

[24] Hudak PL, Amadio PC, Bombardier C, Beaton D, Cole D, Davis A, et al. Development of an upper extremity outcome measure: the DASH (disabilities of the arm, shoulder, and head). Am J Ind Med. 1996;29(6):602–8. 8773720 10.1002/(SICI)1097-0274(199606)29:6<602::AID-AJIM4>3.0.CO;2-L

[25] Franchignoni F, Vercelli S, Giordano A, Sartorio F, Bravini E, Ferriero G. Minimal clinically important difference of the disabilities of the arm, shoulder and hand outcome measure (DASH) and its shortened version (QuickDASH). J Orthop Sports Phys Therapy. 2014;44(1):30–9. 10.2519/jospt.2014.4893 24175606

[26] Földhazy Z, Törnkvist H, Elmstedt E, Andersson G, Hagsten B, Ahrengart L. Long-term outcome of nonsurgically treated distal radius fractures. J Hand Surg. 2007;32(9):1374–84. 10.1016/j.jhsa.2007.08.019 17996772

[27] Young BT, Rayan GM. Outcome following nonoperative treatment of displaced distal radius fractures in low-demand patients older than 60 years. J Hand Surg. 2000;25(1):19–28. 10.1053/jhsu.2000.jhsu025a0019 10642469

[28] Giannoudis P, Tzioupis C, Papathanassopoulos A, Obakponovwe O, Roberts C. Articular step-off and risk of post-traumatic osteoarthritis. Evidence today. Injury. 2010;41(10):986–95. 20728882 10.1016/j.injury.2010.08.003

[29] Lameijer C, Ten Duis H, Vroling D, Hartlief M, El Moumni M, Van der Sluis C. Prevalence of posttraumatic arthritis following distal radius fractures in non-osteoporotic patients and the association with radiological measurements, clinician and patient-reported outcomes. Arch Orthop Trauma Surg. 2018;138:1699–712. 30317380 10.1007/s00402-018-3046-2 PMC6224009

[30] Marchewka J, Szczechowicz J, Marchewka W, Golec E. Long-term outcomes and complications associated with operative and nonoperative treatment of distal radius fractures. Do we need to restore anatomy to have satisfactory clinical outcome? Folia Med Cracov. 2021;61(1). 34185766

[31] Synn AJ, Makhni EC, Makhni MC, Rozental TD, Day CS. Distal radius fractures in older patients: is anatomic reduction necessary? Clin Orthop Relat Research^®^. 2009;467(6):1612–20. 10.1007/s11999-008-0660-2 PMC2674164 19082864

[32] Lee C-H, Kwon Y, Jung IY, Lee B-G, Kim SJ. Effect of the articular surface incongruency on surgical outcome of the distal radius fracture. Biomed Res Int. 2022;2022(1):8357675. 35309177 10.1155/2022/8357675 PMC8926485

[33] Mulders MA, Detering R, Rikli DA, Rosenwasser MP, Goslings JC, Schep NW. Association between radiological and patient-reported outcome in adults with a displaced distal radius fracture: a systematic review and meta-analysis. J Hand Surg. 2018;43(8):710–9. e5. 10.1016/j.jhsa.2018.05.003 29908929

[34] Dankelman LH, Barvelink B, Verhofstad MH, Wijffels MM, Colaris JW. Traditional radiography versus computed tomography to assess reduced distal radius fractures. Eur J Trauma Emerg Surg. 2024;50(5):2313–21. 38985187 10.1007/s00068-024-02598-5 PMC11599432

[35] Meesters AM, Ten Duis K, Banierink H, Stirler VM, Wouters PC, Kraeima J, et al. What are the interobserver and intraobserver variability of gap and stepoff measurements in acetabular fractures? Clin Orthop Relat Research^®^. 2020;478(12):2801–8. 10.1097/CORR.0000000000001398 PMC7899427 32769535

[36] Roelofs LJ, Meesters AM, Assink N, Kraeima J, Van der Meulen TD, Doornberg JN, et al. A new quantitative 3D gap area measurement of fracture displacement of intra-articular distal radius fractures: reliability and clinical applicability. PLoS ONE. 2022;17(9):e0275206. 36166437 10.1371/journal.pone.0275206 PMC9514643

[37] Assink N, Bosma E, Meesters AM, van Helden SH, Nijveldt RJ, Ten Duis K, et al. Initial and residual 3D fracture displacement is predictive for patient-reported functional outcome at Mid-term Follow-Up in surgically treated tibial plateau fractures. J Clin Med. 2023;12(18):6055. 37762994 10.3390/jcm12186055 PMC10531969

[38] Assink N, Kraeima J, Meesters AM, El Moumni M, Bosma E, Nijveldt RJ, et al. 3D assessment of initial fracture displacement of tibial plateau fractures is predictive for risk on conversion to total knee arthroplasty at long-term follow-up. Eur J Trauma Emerg Surg. 2023;49(2):867–74. 36264307 10.1007/s00068-022-02139-y PMC10175438

