# Supplementary material for: Long-term patient-reported outcomes after nonoperative treatment of distal radial fractures: what CT-based gaps and step-offs can be accepted?
Source: Eur J Trauma Emerg Surg. 2025 Sep 9;51(1):291. doi: 10.1007/s00068-025-02954-z (PMC12420767; doi:10.1007/s00068-025-02954-z)
Supplement: Supplementary file 1 — (DOCX 271 KB) [file 68_2025_2954_MOESM1_ESM.docx]

**Supplementary Information:**

**Information manuscript:** Long-term Patient-reported Outcomes after Nonoperative Treatment of Distal Radial Fractures: What CT-based Gaps and Step-offs can be Accepted?, European Journal of Trauma and Emergency Surgery, Lisanne J.M. Roelofs MD*, Tim D. Van der Meulen MD*, Kaj ten Duis MD, Sven H. van Helden MD, PhD, Arvid V.E. Munzebrock MD, Eelke Bosma MD, Job N. Doornberg MD, PhD, Joep Kraeima MD, PhD, Jesse B. Jupiter MD, Jean-Paul P.M. De Vries MD, PhD, Nick Assink MSc, PhD, Frank F.A. IJpma MD, PhD, University Medical Center Groningen, Department of Surgery, Division of Trauma Surgery, Groningen, The Netherlands, f.f.a.ijpma@umcg.nl.

**Table 4** Full regression model of the association between DASH and gap, and DASH and step-off


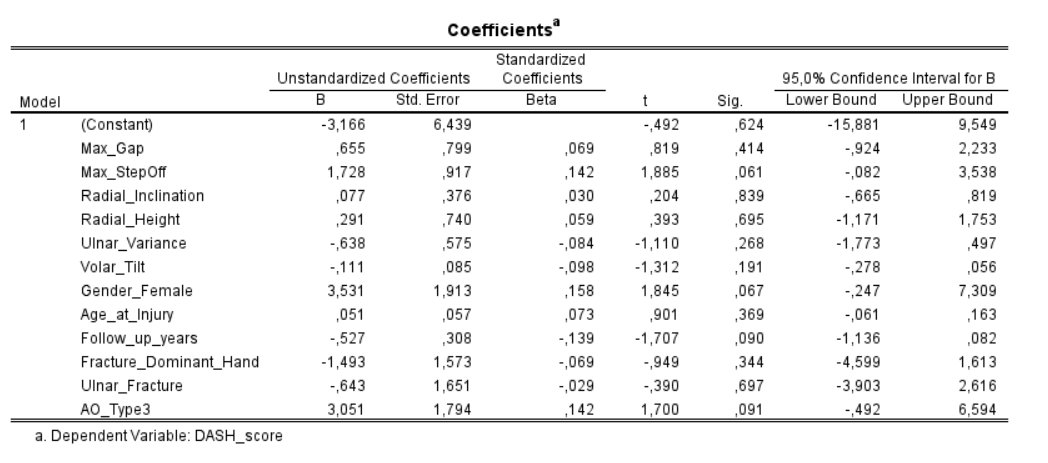


**Table 5** The model summary.


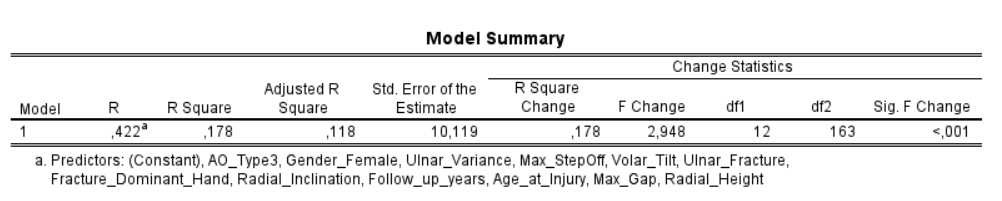


| Table 6: Differences in patient and fracture characteristics between level 1 and level 2 trauma centers | | | |
| --- | --- | --- | --- |
| Level | 1 (N=123) | 2 (N=53) | Sig. (p=) |
| Age Years ± SD* | 55 ± 16 | 52 ± 15 | 0.154 |
| Gender: male | 47 (38%) | 17 (32%) | 0.439 |
| Side of fracture: left | 63 (51%) | 26 (49%) | 0.793 |
| Dominant hand (%) | 62 (50%) | 26 (49%) | 0.870 |
| AO classification (N) |  |  | 0.565 |
| 23B1 | 7 | 1 |  |
| 23B2 | 3 | 1 |  |
| 23B3 | 2 | 0 |  |
| 23C1 | 37 | 18 |  |
| 23C2 | 22 | 6 |  |
| 23C3 | 52 | 27 |  |
| Gap (mm) ± SD* | 2.3 ± 1.2 | 2.3 ± 1.1 | 0.852 |
| Step-off (mm) ± SD* | 0.6 ± 0.9 | 0.7 ± 0.8 | 0.589 |
| Radial inclination (degrees) ± SD* | 21 ± 4 | 23 ± 4 | 0.024 |
| Radial height (mm) ± SD* | 11 ± 2 | 12 ± 2 | 0.091 |
| Ulnar variance (mm) ± SD* | 0 ± 1 | 0 ± 2 | 0.491 |
| Volar tilt (degrees)± SD* | -2 ± 8 | 1 ± 10 | 0.016 |
| Follow-up (years) ± SD | 7 ± 3 | 7 ± 3 | 0.654 |

^*^*^SD=standard deviation^*
